# Stress-induced differential gene expression in cardiac tissue

**DOI:** 10.1038/s41598-021-88267-8

**Published:** 2021-04-28

**Authors:** Ana Elisa T. S. de Carvalho, Marco A. Cordeiro, Luana S. Rodrigues, Daniela Ortolani, Regina C. Spadari

**Affiliations:** Laboratory of Stress Biology, Department of Biosciences, Institute of Health and Society, Campus Baixada Santista, Federal University of São Paulo (UNIFESP), Rua Silva Jardim,136, sala 310, Santos, São Paulo, 11020-015 Brazil

**Keywords:** Cardiovascular diseases, Cardiovascular genetics, Microarray analysis, Signal processing

## Abstract

The stress response is adaptive and aims to guarantee survival. However, the persistence of a stressor can culminate in pathology. Catecholamines released as part of the stress response over activate beta adrenoceptors (β-AR) in the heart. Whether and how stress affects the expression of components of the intracellular environment in the heart is still, however, unknown. This paper used microarray to analyze the gene expression in the left ventricle wall of rats submitted to foot shock stress, treated or not treated with the selective β_2_-AR antagonist ICI118,551 (ICI), compared to those of non-stressed rats also treated or not with ICI, respectively. The main findings were that stress induces changes in gene expression in the heart and that β_2_-AR plays a role in this process. The vast majority of genes disregulated by stress were exclusive for only one of the comparisons, indicating that, in the same stressful situation, the profile of gene expression in the heart is substantially different when the β_2_-AR is active or when it is blocked. Stress induced alterations in the expression of such a large number of genes seems to be part of stress-induced adaptive mechanism.

## Introduction

The stress response is characterized by the activation of the sympathetic nervous system—adrenal medulla, which releases catecholamines, as well as the activation of the hypothalamic–pituitary–adrenal axis, which increases the secretion of glucocorticoids. Catecholamines and glucocorticoids, the hallmarks of the stress response, play fundamental roles as physiological regulators in an attempt to maintain homeostasis and adapt to a new condition. The persistence of the stressful situation may even culminate in increased susceptibility to certain diseases. High levels of catecholamines overstimulate adrenoceptors in the cell membrane of almost every organ, including the β-adrenoceptors (β-AR) in the cardiomyocytes^1^.

In the human and rodent heart, the main β-AR subtype expressed is β_1_-AR over β_2_-AR in a proportion of 80:20^2–4^. Both, β_1_- and β_2_-AR couple to G stimulatory (Gs) protein, which stimulates adenylyl cyclase (AC) to convert ATP in 3′–5′-cyclic adenosine monophosphate (cAMP), which then activates protein kinase A (PKA)^1^. Targets of PKA include L-type calcium channels, T troponin and phospholamban^4^. The phosphorylation of these proteins contributes an increase in the beating rate, development of tension, and the velocity of relaxation of the cardiomyocytes, thus increasing cardiac output. β_2_-AR may also couple to G inhibitory (Gi) protein, with effects opposite to those of Gs stimulation. β_2_-AR-Gi also activates the phosphatidylinositol 3-kinase (PI3K)-Akt signaling pathway, which controls the life and death of cardiomyocytes^5^.

Several studies have already shown the effects of stress in triggering alterations in the cardiovascular system^6–13^. Such alterations can also trigger the onset of diseases, such as atherosclerosis, coronary diseases^12,14^, hypertension, and heart failure^11,12^. Our research group has invested substantial efforts in the investigation of the effects of stress on cardiac reactivity to β-AR stimulation in animal models of stress, mainly the foot shock stress model^10,13^. We have reported that isolated atria of rats submitted to foot shock stress show altered sensitivity to the chronotropic and inotropic effects of catecholamines, which is associated with a remodeling of the proportion of β-AR subtypes in the heart. Isolated atria were subsensitive to β_1_-AR agonists and supersensitive to both β_2_-AR selective agonists and isoprenaline, a non-selective β-AR agonist. These effects of stress were cancelled in the presence of selective β_2_-AR antagonists or non-selective β-AR antagonists, suggesting an increase in the β_2_-AR subtype in the cardiac tissue^10,13,15,16^. A Western blot approach confirmed the higher expression of β_2_-AR accompanied by a low expression of β_1_-AR in the atrium and ventricle of rats submitted to foot shock stress when compared to control non-stressed rats^13^. A reduced cardiac β_1_/β_2_-AR ratio is also involved in other circumstances, such as aging and heart failure. It is considered adaptive since it protects the heart from the cardiotoxic effects of persistent β_1_-AR overstimulation^1,5,17,18^.

In contrast to the recognized cardiotoxic effect of persistent β_1_-AR stimulation, the possible cardioprotective role of prolonged β_2_-AR activation is still controversial. After the receptor couples with Gi, the Giα subunit inhibits AC, attenuating the signal mediated by β_1_-AR, while the Giβγ activates several intracellular signals, including PI3K-Akt^19,20^. Despite the beneficial effect on the viability of cardiac cells, β_2_-AR stimulation compromises contractility. Recently, we have demonstrated that the activity of PI3K-Akt is reduced in the heart of foot shock stressed rats even 5 days after the last stress session^21^. The persistence of this effect indicates the relevance of the intracellular processes triggered by stress in the heart. Moreover, the increase in β_2_-AR participation in the cardiac stress response and heart failure suggests an important role for β_2_-AR signaling in cardiac events. However, the complex biological processes contributing to the cardiac response to stress are not yet fully understood.

To contribute to our understanding of the influence of stress on cardiac physiology, we have proposed an evaluation of the gene expression profile in the cardiac tissue of rats submitted to foot shock stress. We have also compared the gene expression profile in the presence of stress-induced upregulation of β_2_-AR and in the absence of β_2_-AR signaling due to the pharmacological blockade of the receptor. Our results provide insight into the role played by β_2_-AR in the differential regulation of gene expression during stress.

## Results

**Figure 1:**
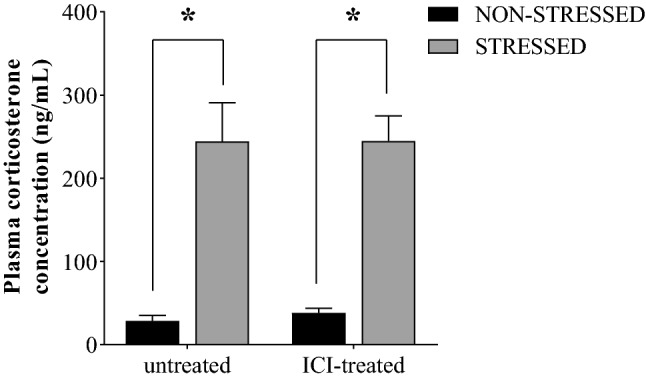
Plasma concentration of corticosterone in untreated non-stressed control rats, untreated rats submitted to stress, non-stressed rats treated with ICI118,551, and rats both treated with ICI118,551 and submitted to stress. The bars indicate mean ± S.E.M. of 6 rats/group. *p ≤ 0.05 compared to control, two-way ANOVA plus Tukey test.

The corticosterone plasma level was higher in the rats submitted to foot shock stress than in non-stressed rats. The β_2_-AR blockade with ICI118,551 did not interfere with this endocrine stress response (Fig. 1).

### β_2_-AR drives gene expression in the heart post stress

In order to identify the genes of which expression was altered in the heart of rats submitted to stress, samples were arranged for microarray so that untreated rats with and without stress were compared. Since β_2_-AR is upregulated in the heart of stressed rats^13,21^, its influence on target genes expression under stress was also evaluated with ICI treatment (with or without stress).

In microarray analysis, 265 genes were found to be significantly disregulated in non-ICI treated rats, with 208 upregulated and 57 downregulated under stress. β_2_-AR blockade resulted in the significant disregulation of 114 genes under stress, with 90 being upregulated and 14 downregulated. The functional analysis of the genes with differential expression was performed using IPA software. Interestingly, many non-annotated genes were disregulated in both comparisons, but mainly those in the comparison of the untreated groups (Table 1, Supplementary Tables S1, S2).

**Table 1:** Most disregulated genes identified in the heart by the Ingenuity Pathway Analysis based on the comparison of stressed with non-stressed untreated rats (4 rats/group), and those stressed and unstressed treated with ICI118,551 (4 rats/group).

	Untreated		ICI-treated	
	Gene	Log ratio	Gene	Log ratio
	LOC102S56673*	3.327	HSPA1A/HSPA1B	3.867
	LOC102551486*	2.572	APOLD1	2.195
	RGS1	2.489	IRS2	2.132
	PDLIM3	2.168	Sik1	2.041
UP	LOC103691S66*	2.12	PER1	2.016
	EGR1	2.087	TRIM21	2.029
	LOC103693175*	1.966	USP2	1.907
	CREM	1.933	HSP90AA1	1.836
	IL6R	1.923	STC1	1.828
	LOC100909409*	1.909	ATF3	1.814
	MYCN	− 2.039	SEZ6L	− 2.84
	C2CD4A	− 1.634	CCL2	− 2.171
	CDKN2C	− 1.554	SGCB	− 1.904
	GSTA5	− 1.534	Hist1h1b	− 1.684
DOWN	PTGDS	− 1.458	PM20D2	− 1.341
	RRAD	− 1.41	KIF22	− 1.319
	TLR3	− 1.402	APLNR	− 1.31
	MYBPC2	− 1.399	LOC102548611*	− 1.277
	TTC30B	− 1.366	ARNTL	− 1.238
	IFi47	− 1.307	ART5	− 1.146

The disregulated genes were ranked according to experimental log ratio. Thresholds were based on extent of fold change (≥ 2) and p value ≤ 0.05.

The lists with additional information are available in Supplementary Tables S1 and S2.

*Non-annotated genes.

The genes with the greatest differential expressions are listed in Table 1. In the comparison of untreated rats, the genes RGS1 (regulator of G protein signaling), PDLIM3 (PDZ and LIM domain 3), EGR1 (early growth response 1), CREM (cAMP) responsive element modulator) and IL6R (interleukin 6 receptor) underwent the greatest upregulation in the presence of stress, while MYCN (MYCN proto-oncogene), TLR3 (toll like receptor 3) and MYBPC2 (myosin binding protein C2) were downregulated.

For the comparison of the ICI-treated groups (with versus without stress), the greatest upregulation was found for the genes coding heat shock proteins (HSPA1A/HSPA1B and HSP90AA1), insulin receptor substrate 2 (IRS2) and ATF3 (activating transcription factor 3), whereas SEZ6L (seizure related 6 homolog like), CCL2 (C–C motif chemokine ligand 2) and APLNR (apelin receptor) underwent downregulation (Table 1).

**Figure 2:**
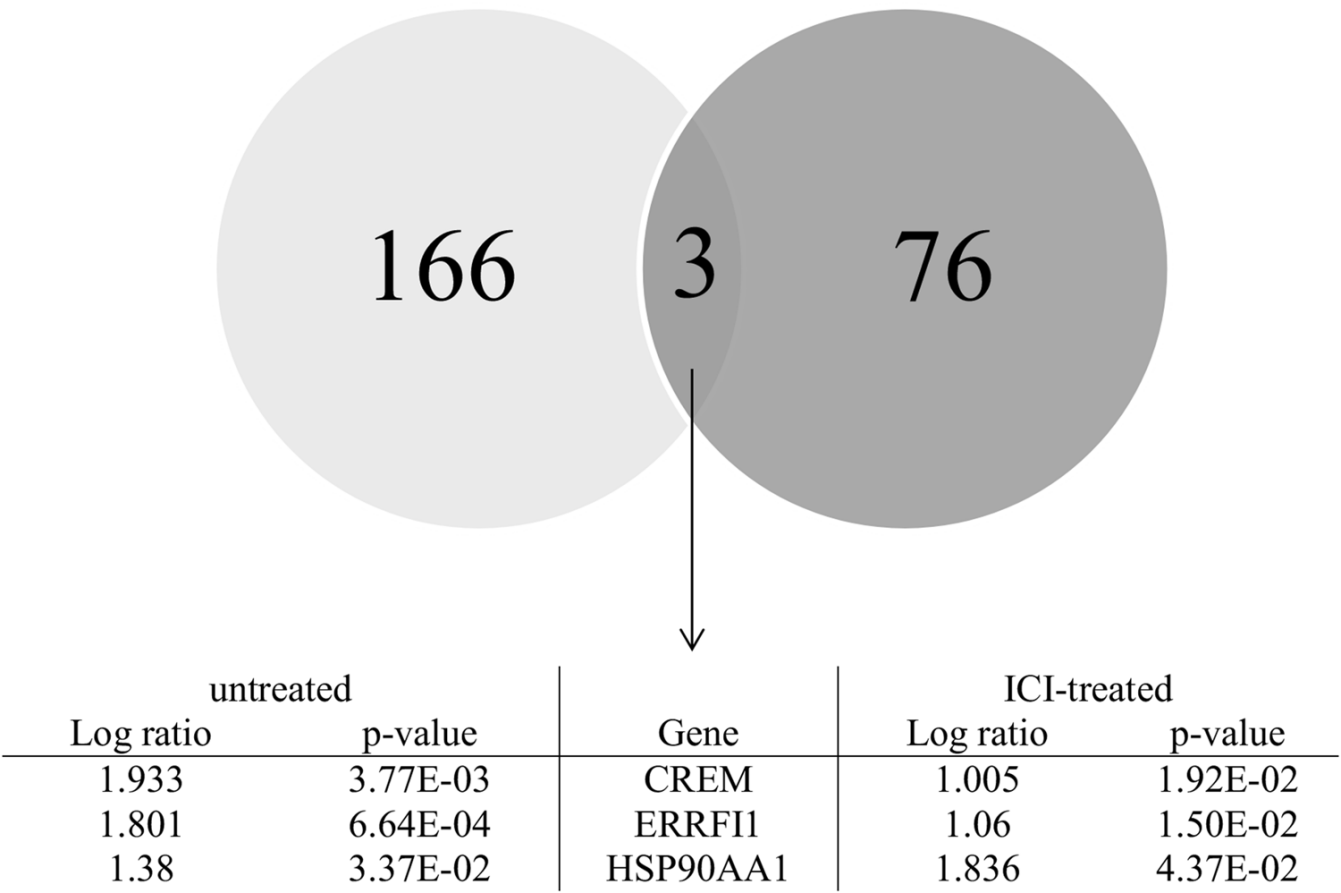
Venn diagram showing the number of disregulated genes by stress based on the comparison of stressed with non-stressed untreated rats (light gray; 4 rats/group), and those stressed and unstressed treated with ICI118,551 (dark gray; 4 rats/group). Only three genes were affected in the two comparisons. The list of affected genes includes name, location, type, p value, and log ratio.

There was minimal overlap in the genes disregulated by stress in the two comparisons, as can be seen in the Venn diagram (Fig. 2). Three genes proved to be upregulated under stress, independent of the presence of the β_2_-AR: cAMP responsive element modulator (CREM), ERBB receptor feedback inhibitor 1 (ERRFI1), and heat shock protein 90 alpha family class A member (HSP90AA1). The vast majority of disregulated genes were specific for one of the conditions, with 166 annotated genes being disregulated in cardiac tissue when β_2_-AR was active (ICI-untreated), and 76 when β_2_-AR was blocked (ICI-treated).

### Stress induces alterations in the expression of genes related to inflammatory response, cell cycle and proliferation

**Figure 3:**
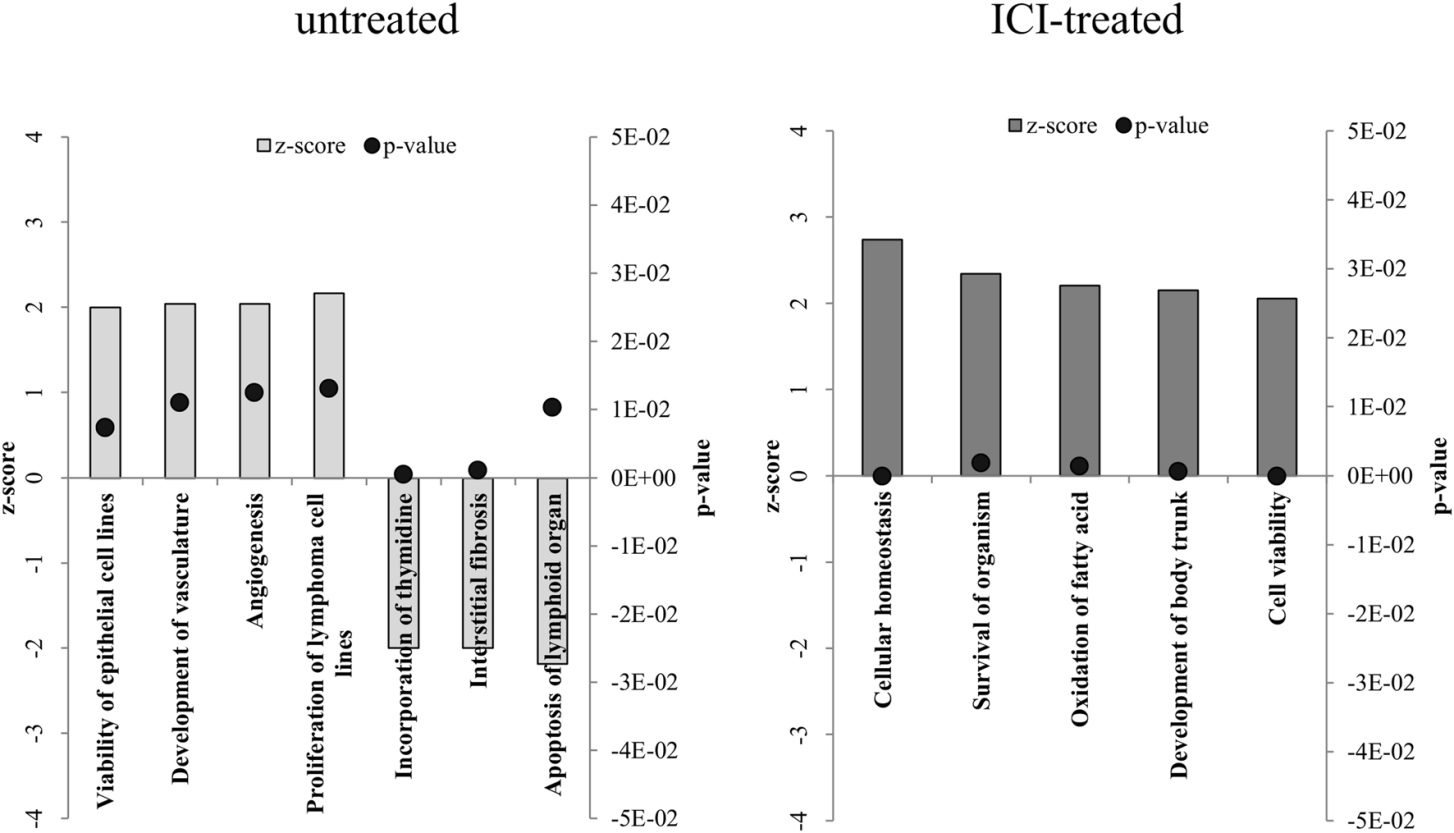
Diseases and functions identified in the heart by the Ingenuity Pathway Analysis based on the comparison of stressed with non-stressed untreated rats (4 rats/group), and those stressed and unstressed treated with ICI118,551 (4 rats/group). Bars indicate z-score, and black dots indicate the p value for each category. The thresholds were a z score of ≥ 2 or ≤ − 2 and p value of ≤ 0.05. A list with additional information is available in Supplementary Table S3.

An analysis of the disregulated genes suggests that certain categories of diseases and functions may be triggered or altered in the cardiac tissue of rats submitted to stress (Supplementary Table S3). Figure 3 shows some of these categories of diseases and altered function. In the comparison of the non-ICI treated groups, stress enhanced the expression of genes encoding by proteins related to angiogenesis and development of vasculature, epithelial cell viability, and proliferation of lymphoma cells, while reducing that of genes encoding proteins related to incorporation of thymidine, interstitial fibrosis and apoptosis of lymphoid organs. In ICI-treated groups, the presence of stress increased the expression of genes associated with homeostasis, survival, and the oxidation of fatty acids (Fig. 3).

The analysis of canonical pathways allows visualization of the signaling pathway in which the significantly disregulated genes are inserted, as well as their functional relevance. In the non-treated groups’ comparison, no canonical pathway was activated, although pathways related to the inflammatory response, regulation of cell cycle and proliferation were listed (Table 2), with the most frequently affected genes in these pathways being ALAD (aminolevulinate dehydratase), CPOX (coproporphyrinogen oxidase), CDK1 (cyclin dependent kinase 1), HSP90AA1 (heat shock protein 90 alpha 1) and HDAC8 (histone deacetylase 8) (Supplementary Table S4). Among the canonical pathways activated in the comparison of stressed and unstressed ICI-treated rats, the acute phase response signaling and IL6 signaling were positively regulated, while cAMP mediated signaling was negatively regulated (Table 2). Other pathways were listed including those associated with the Th17 inflammatory response, the regulation of the immune response, glucocorticoid and mineralocorticoid signaling, and cAMP signaling. Interestingly, most of pathways shared the same set of disregulated genes, including JUN (JUN proto-oncogene), MAP2K3 (mitogen-activated protein kinase 3), NFKBIA (NFκB inhibitor alpha), CEBPB (CCAAT enhancer binding protein beta), HSP90AA1 (heat shock protein 90 alpha 1), HSPA1A/HSPA1B (heat shock protein family A -Hsp70-members 1A and 1B), and DUSP1 (dual specificity phosphatase 1) (Supplementary Table S4).

**Table 2:** Canonical pathways identified in the heart by the Ingenuity Pathway Analysis based on the comparison of stressed with non-stressed untreated rats (4 rats/group), and those stressed and unstressed treated with ICI118,551 (4 rats/group).

Untreated	ICI-treated
Heme biosynthesis II	Glucocorticoid receptor signaling
Eicosanoid signaling	IL17A signaling in fibroblasts
Mitotic roles of polo-like kinase	IL17 signaling
Cyclins and cell cycle regulation	Circadian rhythm signaling
S-adenosyl-L-methionine biosynthesis	Protein ubiquitination pathway
Cell cycle regulation by BTG family proteins	**Acute phase response signaling**
Heme biosynthesis from uroporphyrinogen III I	Apelin endothelial signaling pathway
Spermine and Spermidine degradation I	**IL6 signaling**
Tetrapyrrole biosynthesis II	PI3K signaling in B lymphocytes
Telomerase signaling	CD27 signaling in lymphocytes
	CD40 signaling
	IL10 signaling
	*cAMP mediated signaling*

Bold shading indicates prediction of activation and italics indicates prediction of inhibition.

A list with additional information is available in Supplementary Table S4.

### Upstream molecules triggering gene expression changes

The IPA analysis of upstream regulators uses a knowledge data base to predict the upstream molecules that could be triggering the experimentally observed gene expression changes. This analysis suggested that the extra-cardiac environment was strikingly different in rats submitted to stress whether or not they had been treated with the β_2_-AR blocker (Table 3, Supplementary Table S5). Table 3 shows the most altered upstream molecules. GPER1 (G protein-coupled estrogen receptor 1) was predicted to be an upstream regulator in the presence of stress, independent of ICI-treatment. It leads to AC activation, thus increasing cAMP levels. Very few upstream inhibitory regulators were identified (Table 3). The transcription factor CREB1 (cAMP responsive element binding protein 1) was the most altered upstream regulator for the comparison of ICI-treated groups (Table 3) but it was also present in the comparison of the untreated groups (Supplementary Table S5). CREB1 is the transcription factor that binds CREM as a response to an increase in cAMP levels. Among the upstream regulators with specific relevance in the comparison of non-ICI treated groups were pro-inflammatory cytokines, IL1B (interleukin 1 beta) and TNF (tumor necrosis factor), as well as calcium (Table 3), while the specific upstream regulators in the comparison of ICI-treated groups were insulin and norepinephrine (Table 3).

**Table 3:** Most altered endogenous upstream regulators identified in the heart by the Ingenuity Pathway Analysis based on the comparison of stressed with non-stressed untreated rats (4 rats/group), and those stressed and unstressed treated with ICI118,551 (4 rats/group).

Upstream regulator	Type	Predicted state	z score	p value
**Untreated**				
TNF	Cytokine	Activated	3.088	7.32E−07
IL1B	Cytokine	Activated	2.967	8.07E−10
F2	Peptidase	Activated	2.757	4.20E−04
CG	Complex	Activated	2.701	4.00E−05
Ca^+2^	Chemical-endogenous	Activated	2.586	1.80E−03
GPER1	G-protein coupled receptor	Activated	2.425	8.28E−07
MAPK1	Kinase	Activated	2.414	2.60E−03
MAPK8	Kinase	Activated	2.391	9.69E−04
SMARCA4	Transcription factor	Activated	2.213	4.45E−02
ACOX1	Enzyme	Inhibited	− 2.0	1.87E−02
**ICI-treated**				
CREB1	Transcription factor	Activated	3.634	1.40E−12
Insulin	Group	Activated	3.035	1.15E−15
NUPR1	Transcription factor	Activated	3.0	6.56E−05
PGR	Ligand-dependent nuclear	Activated	2.96	6.53E−09
GPER1	G-protein coupled receptor	Activated	2.646	3.89E−10
HIF1A	Transcription factor	Activated	2.574	7.44E−04
Norepinephrine	Chemical-endogenous	Activated	2.553	1.43E−12
Hydrogen peroxide	Chemical-endogenous	Activated	2.528	8.19E−10
EGE	Growth factor	Activated	2.459	2.17E−08
NKX2-3	Transcription factor	Inhibited	− 2.0	6.88E−03

The thresholds were z score (≥ 2 or ≤ -2) and p value (≤ 0.05).

The list with additional information is available in Supplementary Table S5.

## Discussion

The data presented here were obtained by microarray technology that provided an overview of the level of mRNA in the heart of rats submitted to stress, treated or not treated with a β_2_-AR antagonist. It was assumed that the level of mRNA reflects gene expression even though it may suffer post translational modifications, a process that might also be altered by stress, as it was previously reported^22^.

It has been shown that stress induces changes in gene expression and that β_2_-AR modulates those changes in the heart. The vast majority of genes with expression disregulated by stress were different when β_2_-AR was upregulated and when it was blocked by the ICI treatment; only three genes were disregulated in both cases. This indicates that, given the same stressful situation, the profile of gene expression in the heart is substantially different when β_2_-AR is active or when it is blocked. The three genes with expression independent of β_2_-AR were CREM, which is related to the cAMP signaling pathway, HSP90AA1, which encodes HSP90, the glucocorticoid receptor chaperone, and ERRFI1, which encodes the feedback inhibitor of the epidermal growth factor receptor, which attenuates the PI3K-Akt signaling pathway.

### Effect of stress on genes related to cAMP signaling pathway

β-AR activation by catecholamines is the main signal for cAMP generation in cardiac myocytes. The cAMP-dependent transcription factor, CREM, expressed in the myocardium, is involved in the regulation of the expression of various components of the cAMP signaling pathway^23^. It has been described as essential for normal cardiac function^24,25^ and required for the β_1_-AR response to overstimulation^25^. CREM upregulation in the heart of stressed rats probably contributes for the efficacy of β_1_-AR (with or without the participation of β_2_-AR) signaling in the context of overstimulation. Moreover, many other genes involved in that same signaling pathway had their expression disregulated under stress, whether or not treated with ICI118,551. RGS1 and RGS16 were upregulated in the untreated groups’ comparison. The RGS family acts as a negative regulator of G protein signaling. By controlling heterotrimeric G proteins they may regulate myocardial hypertrophy and contractility^26^. RGS1 has been related to the control of inflammation and the immune response^27,28^. The stress-induced alteration in the expression of these genes depends on an active β_2_-AR, since it is unaltered in the group treated with ICI118,551. On the other hand, CREB1 and GPER1 belong to the group of molecules predicted to be upstream regulators activated by stress, independent of β_2_-AR activation. CREB1, a transcription factor responsive to cAMP, leads to effects similar to CREM^24^, while GPER1 belongs to the G-protein coupled receptor family that binds estrogen. This receptor activates both the adenylate cyclase-cAMP/PKA signaling pathway ^29,30^ and the PI3K-Akt-mTOR signaling pathway^31^. These two signaling pathways are related to cardioprotection and cell survival^20^.

The APLNR gene, also called the APJ receptor gene, encodes the G-protein coupled receptor for apelin, a protein expressed in the cardiovascular system which promotes a positive inotropic effect on the heart as well as angiogenesis and blood vessel relaxation^32,33^. Apelin not only increases inotropy, but also decreases left ventricular pre- and afterload due to its pronounced vasodilation effect^34^. The inotropic action of apelin is the result of an increase in the availability of intracellular calcium^35^. It also induces cAMP synthesis, as well as activating the PI3K-Akt signaling pathway^33^. The concentration of apelin is reduced in the failing heart^36^ and the contractile function is impaired in cardiomyocytes with knockout for APLNR^37^. Apelin expression was downregulated under stress when no ICI118,551 treatment was given, although the expression of its receptor (APLNR) was reduced when the β_2_-AR was blocked.

Therefore, in the heart of untreated stressed rats, the downregulation of β_1_-AR and apelin seems to be counterbalanced by the upregulation of β_2_-AR and the action of CREM. Hence, although the proportion of β-AR subtypes is altered, there is no difference in cAMP formation by left atrial membranes of control and foot shock stressed rats stimulated by non-selective agonists^13^. However, when the membranes are stimulated in the presence of the β_2_-AR antagonist (ICI118,551), the amount of cAMP synthetized by the atrial membranes of stressed rats is lower than that of unstressed ones^13^. This is probably because the increase in the expression of CREM is not sufficient to sustain the synthesis of cAMP, since β_1_-AR is downregulated, as has been reported elsewhere^13^.

The present data thus confirm that when stress is applied under conditions of β_2_-AR blockade, the canonical pathway of cAMP signaling is negatively regulated (see Table 2), due largely, but not only, to β_1_-AR and APLNR downregulation. The increasing cAMP level due to β_1_-AR and β_2_-AR activation culminates in an increased rate of beating and force developed by isolated left atrium^13^. The calcium transient plays a central role in this process^38^. Indeed, calcium was predicted to be an upstream regulator in the presence of higher β_2_-AR expression. Although extremely important for the proper functioning of cardiac cells, excessive calcium leads to disorders such as arrhythmia, hypertrophy, and cell death^38^. Therefore, the stress induced modulation of positive and negative influences on the expression of molecules related to cAMP and the calcium signaling pathways in the heart adds complexity, as well as more possibility for control of the cardiac function and structure, with β_2_-AR apparently playing an essential role in the process.

### Effect of stress on glucocorticoids signaling

Glucocorticoids (GC) and catecholamines are known as the stress hormones. The glucocorticoid receptor (GR) is located in the cytoplasm of target cells, bound to a chaperone complex and immunophilins that provide structural stability and function^39^. Upon the binding of the GC, the GR undergoes a conformational change that causes the release of the chaperones and dimerization of the complexes GC–GR. Then, the GC–GR dimers translocate to the nucleus, where they start its genomic signaling^40,41^. Increased corticosterone plasma levels led to an increase in GR-mediated genomic signaling and the modulation of gene expression.

The heat shock proteins (HSP) 90 and 70, as well as the proto-oncogene tyrosine-protein kinase (Src), released from the GR complex, also influence cell signaling^41^ by regulating protein folding, proteostasis and intracellular signal transduction. HSP90 and HSP70 are inducible isoforms that present incremented expression after myocardium injury, oxidative stress, and hypoxia. HSP90aa1, the mRNA for HSP90, was upregulated under stress, both with and without β_2_-AR participation, while HSPa1a, the mRNA for HSP70, was upregulated by stress only under β_2_-AR blockade. Upregulation of HSP90 and HSP70 has been linked to cardioprotection against injury^39,42,43^. The in vitro overexpression of HSP90aa1 in cardiomyocytes attenuates apoptosis by increasing Bcl-2 expression^43^. HSP90 is also associated with the survival pathway of PI3K-Akt. The association of HSP90 with Akt leads to phosphorylation and the activation of endothelial nitric oxide synthase^39,42^. Under stress, in the presence of β_2_-AR upregulation, the reduction of fibrosis and apoptosis that was indicated by disease and function categorization, suggests that the modulation of the expression of genes is part of the adaptive mechanisms to stress.

The activation of GR signaling is also related to the induction of the expression of the gene ERRFI1. This gene encodes a feedback inhibitor of the epidermal growth factor receptor (EGFR) what reduces cardiac hypertrophy^44,45^. The results presented here have demonstrated that in rats submitted to stress as compared to non-stressed rats, the circulating levels of corticosterone, the predominant GC in rodents, were higher and the ERRFI1 gene expression was upregulated, whether β_2_-AR is upregulated or blocked. Therefore, a reduction of Akt phosphorylation is expected. Accordingly, a reduced Akt phosphorylation has been reported in the ventricles of rats submitted to the same stress protocol used here^21^.

**Figure 4:**
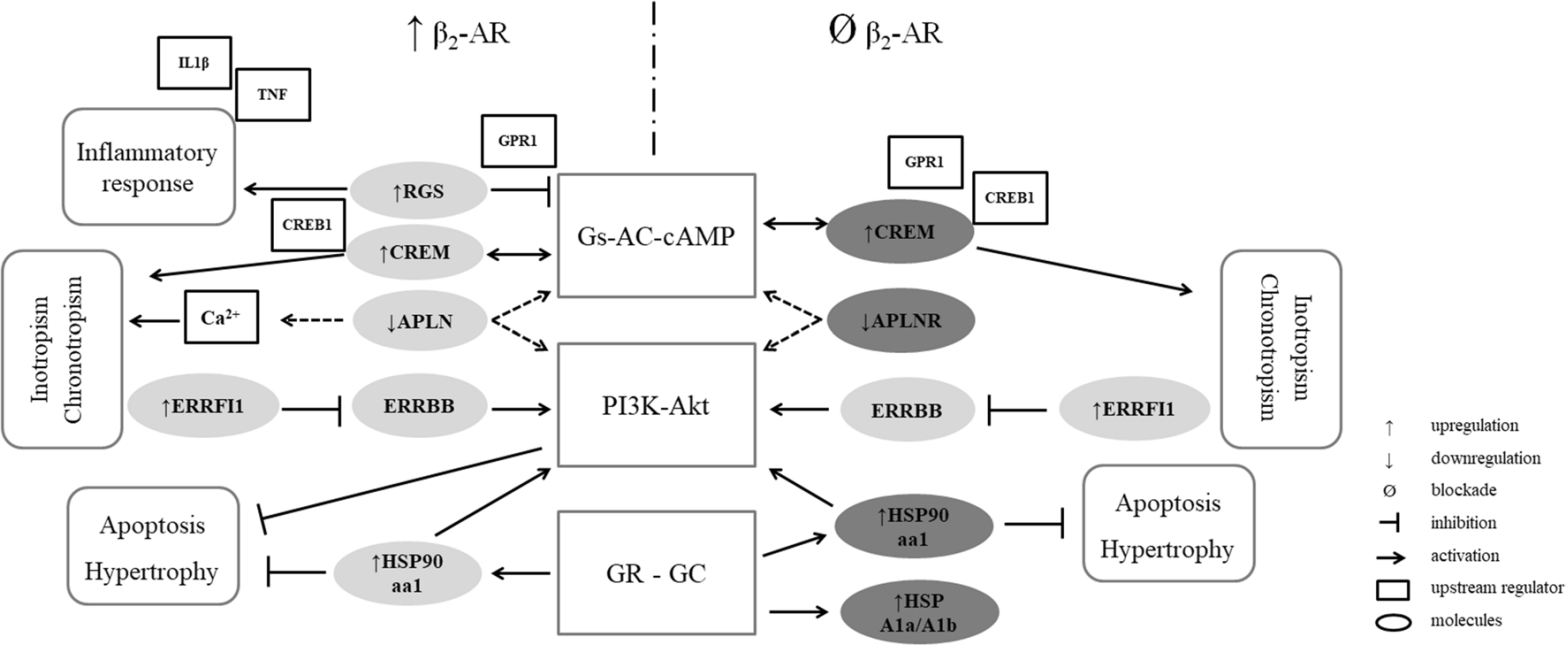
Schematic representation of the microarray analysis of the effect of stress on the expression of most disregulated genes in the ventricle of rats with β_2_-adrenoceptors (β_2_-AR) upregulated (unstressed and stressed untreated rats comparison; left side) and those with the β_2_-AR antagonized by ICI118,551 (comparison of unstressed and stressed ICI-treated rats; right side). The signaling pathways related to these genes are listed in the center of the figure, and the putative effect on cardiac function and structure are listed in the respective side of each condition.

The present data have thus suggested that two signaling pathways are the most affected by stress: the β-AR-Gs-AC-cAMP and the PI3K-Akt signaling pathways. Moreover, the modulation of several of their components depends on the presence of the β_2_-AR. The changes induced by stress in the β-AR-Gs-AC, GC-GR, and PI3K-Akt signaling pathways are summarized in Fig. 4. On the left side are listed some of the genes with regulation altered by stress (upregulation of ERRFI1, CREM, RGS1, HSP90aa1; and downregulation of APLN) and some predicted upstream molecules. The right side of Fig. 4 shows the genes that are altered and predicted upstream molecules when stress is applied in conjunction with the β_2_-AR blockade: ERRFI1, CREM, HSP90aa1, and HSPA1A/A1B are upregulated whereas APLNR is downregulated.

The changes post stress in cardiac gene regulation also include the progression of the cell cycle, which can be impaired by downregulation of CDK1, and the presence of epigenetic factors, such as the upregulation of the class I histone deacetylase, HDAC8, and, in ICI-treated rats, stress induced upregulation of HSP40, and IRS2. The upregulation of IRS2 suggests that insulin may be an upstream regulator of the stress response, independent of β_2_-AR, since it appears in the comparisons of both non-ICI treated and ICI treated rats.

### Effect of stress on components of the immune system

An unexpected finding was the recruitment of the immune system to the cardiac tissue of stressed rats, with the profile clearly different for the two groups, as a function of β_2_-AR. Immunological activation was identified in microarray data in expressed genes, canonical pathways and upstream regulators. The correlation of immunological response with cardiac injury and repair is widely recognized in the failing heart^46–48^ and in human coronary heart disease^49–51^.

Among the differently expressed genes in the comparison of non-ICI treated group, IL22RA2 (interleukin 22 receptor subunit alpha 2), IL2RA (interleukin 2 receptor antagonist) and IFRD1 (interferon related developmental regulator 1) were upregulated and are described as regulators of the inflammatory response^52,53^. IL-6 was also upregulated in this group. This is a pro-inflammatory cytokine produced by myeloid cells and cardiomyocytes in an autocrine mechanism^54^, which has been related to cardiac disease and the impairment of cardiac performance^50,51^.

It has been predicted that the pro-inflammatory cytokines IL-1β and TNF act as upstream regulators of the cardiac stress response in the presence of β_2_-AR. Both are related to acute inflammation in cardiac disease, such as heart failure and myocardial infarction^46,54^. IL-1β, TNF-α, and IL-12 are cytokines released by T helper 1 lymphocytes (Th1), which control the cellular immune response. Interestingly, immune cells, including Th1 lymphocytes, express β_2_-AR and are, therefore, susceptible to catecholamines action^55^. In immune cells, cAMP-PKA signaling inhibits the transcription of nuclear factor kappa B (NF-κB); through CREB, it activates the transcription of IL-10. This mechanism promotes the differentiation of the Th2 response, which is markedly anti-inflammatory, and also inhibits the development of the Th1 response, which is markedly pro-inflammatory^55,56^. The chronic use of β_2_-AR-selective and nonselective blockers in mice impairs the recruitment of leukocytes to the injured heart and reduces survival^57^.

The data reported here thus suggest that in the presence of greater expression of β_2_-AR, as previously reported by Moura et al.^13^, a pro-inflammatory signaling is triggered in the heart of stressed rats. If the upregulation of β_2_-AR occurs only in the cardiomyocytes or in the resident immune cells as well is, however, unknown at the present time. Indeed, data for the comparison of stressed ICI-treated rats with non-stressed ICI-treated rats did not involve pro-inflammatory genes, which suggests that the treatment with ICI188,551 reduced the development of the Th1 response by blocking β_2_-AR in the immune cells as well. Several canonical pathways listed in that analysis are related to the immune response, such as the signaling pathway of IL-10, IL-17, CD40, CD27 and IL-6, the acute phase response, and GR signaling pathway. One interesting characteristic of these canonical pathways is the expression of the same gene, NFKBIA, at times in conjunction with MAP2K3 and/or JUN. NFKBIA encodes a potent inhibitor of NF-κB^58^, which mediates activation of the inflammatory response with important consequences in heart disease. The overexpression of NFKBIA in cardiomyocytes inhibits NF-κB activity, reduces hypertrophy and improves cardiac performance and survival via the Akt signaling pathway^59^. The presence of NFKBIA in the left ventricle suppresses the expression of NF-κB and attenuates myocardial fibrosis^60^. Therefore, the expression of NFKBIA mRNA in ICI-treated stressed rats could indicate the presence of a regulatory or anti-inflammatory mechanism in the ventricle of rats submitted to stress when faced with β_2_-AR blockage.

### Study limitations and conclusion

The data has shown that stress induced alterations in the expression of such a large number of genes what seems to be part of adaptive mechanisms. β_2_-AR clearly plays a role in this process, since the alterations in gene expression that occurred in the presence of this adrenoceptor subtype are completely different from those seen when it is blocked. However, it might be considered that there are many processes between the gene expression and the respective protein synthesis and activity. Indeed, one of the major challenges in all experimental systems is the issue of causality and association. Thus, in order to confirm if the altered gene expression here demonstrated has functional consequences, additional experiments are required. The lack of functional experiments and protein validation is a limitation of this work. Despite that, the great number of significant data presented here suggests that might be an influence of stress in the cardiac cells phenotype, and probably in the heart function.

## Methods

### Animals and experimental groups

Male Wistar rats (*Rattus norvegicus*; 24 animals; 250–350 g; 12 weeks-old), obtained from the Center for the Development of Experimental Models (CEDEME), of the Federal University of São Paulo (São Paulo, SP, Brazil), were housed in standard cages in a temperature-controlled room (22 °C) on a 12:12 h light:dark cycle, with the lights on at 7:00 am. Standard laboratory chow and tap water were available ad libitum. The rats were randomly distributed in two groups as follows: untreated and treated with ICI118,551 (ICI), with two subgroups each: submitted to stress and not submitted to stress. The experimental protocols were approved by the Ethics Committee for Animal Use of the Federal University of São Paulo (CEUA/UNIFESP), protocol number 86613101116, in accordance with the Brazilian National Council for Control of Animal Experimentation (CONCEA, Brazil), and all study carried out in compliance with the ARRIVE guidelines.

### Stress protocol

The foot shock stress protocol was administered as previously described^13,61,62^. The rats in the stressed groups (both untreated and ICI-treated; 6 rats/group) were submitted to foot shock sessions; rats in the non-stressed groups (untreated and ICI-treated; 6 rats/group) were also placed in the foot shock cage, but did not receive foot shocks. The stress cage was a Plexiglas chamber (26 × 21 × 26 cm) provided with a floor grid made of stainless-steel rods (0.3 cm in diameter, spaced 1.5 cm apart). During the daily 30 min stress sessions, which occurred between 7:30 am and 11:00 am on three consecutive days, foot shocks were delivered by a constant current source controlled by a microprocessor-based instrument. The intensity of the current was 1.0 mA, with duration of 1.0 s, with pulses delivered at random intervals of between 5 and 25 s. The rats were returned to their standard cages after the first and second period in the Plexigas chamber. After the third session, the rats were immediately euthanized by decapitation, in compliance with the American Veterinary Medical Association (AVMA) Guidelines for the Euthanasia of Animals (2020). The trunk blood was collected in a tube containing EDTA and centrifuged. The plasma was separated and stored at – 80 °C. The hearts were harvested and the left ventricles were isolated and stored at − 80 °C.

### Treatment with ICI118,551

For 5 days, the rats in the ICI-treated groups, stressed or not, received 500 µg/kg/day, i.p, of ICI118,551 ((±)-1-[2,3-(dihidro-7-metil-1H-inden-4-il) oxi]-3-[1-metilletil) amino]-2-butanol; Tocris Bioscience, Bristol, UK), a highly selective β_2_-AR antagonist^63^. The rats in the untreated non-stressed control group and those submitted to stress that were not treated with ICI received injections of saline solution, i.p. The foot shock stress sessions began on the third day of the treatment with ICI or saline solution.

### Plasma corticosterone concentration

The plasmatic concentration of corticosterone was determined in all experimental groups (6 rats/group) by enzyme immunoassay (ELISA) using a commercial kit (Enzo Life Science, Inc., Ann Arbor, MI, EUA) according to the manufacturer's guidelines.

### RNA preparation and processing

Total RNA was extracted from fragments of up to 100 mg from the left ventricle from all experimental groups (4 rats/group) using TRIzol reagent according to the manufacturer’s instructions (Invitrogen, Carlsbad, CA, USA). The final volume of the samples was 100 µL of water treated with 0.1% dietilpyrocarbonate (DEPC UltraPure; Invitrogen, Carlsbad, CA, USA); these were stored at − 80 °C overnight. The mRNA concentration and degree of purity were determined in Nanodrop 2000 c (Thermo Scientific, Waltham, MA, USA) under 260/280 nm. The RNA was purified using an RNeasy Kit (Qiagen, Valencia, CA, USA) according to the manufacturer's guidelines. The purified RNA concentration and degree of purity were determined in Nanodrop 2000 c.

### Microarray assay

The gene expression profile was evaluated for the total RNA isolated from the left ventricles of the rats in each of the experimental groups. The two-color microarray-based gene expression analysis was Agilent-074036 SurePrint G3 Rat GE v2 8 × 60 K Microarray G4858A (GEO GPL22145) from Agilent Technologies (Santa Clara, CA, USA). There were a total of four samples for each group. Each sample was analyzed once. The analysis was performed at the Center of Excellence—Genomics (Agilent Technologies Brasil, Alphaville, Barueri, São Paulo, Brazil).

Firstly, the total RNA samples were assayed using RNA ScreenTape Analysis in a TapeStation System (Agilent Technologies, Santa Clara, CA, USA) to determine RNA quality and concentration (ng/μL). 200 ng of total RNA were used for individual reactions using oligo-dt linked to T7 promoter primer, being the Spike A Mix/Cyanine 3-CTP dye for the non-stressed samples and Spike B Mix/Cyanine 5-CTP dye for the stressed samples. The transcription of cDNA was performed using cDNA Master Mix for 2 h at 40 °C, followed by 15 min at 70 °C. The cDNA served as a template for the next transcription reaction. For the in vitro transcription of cRNA, Cyanine 3-CTP (for the non-stressed samples) and Cyanine 5-CTP (for the stressed samples) were incorporated, using T7 RNA polymerase for 2 h at 40 °C. After this, the samples were stored at − 80 °C. On the following day, the amplified and labeled cRNA was purified using an RNeasy kit, according to the manufacturer's guidelines, and the concentration was determined by RNA ScreenTape Analysis in a TapeStation System. For each microarray, 300 ng portions of each labeled cRNA sample were assembled in pairs of Cyanine 3-CTP (for non-stressed samples) and Cyanine 5-CTP (for stressed samples) incorporation as follows: samples of non-stressed untreated with stressed untreated groups, and samples of non-stressed ICI treated with stressed ICI treated groups. Then they were fragmented for 30 min at 60 °C. The hybridization assembly was prepared according to the Agilent microarray hybridization chamber user guide. The hybridization was performed in the Agilent Microarray Hybridization Quick Assemble Chamber for 17 h at 65 °C at a rotation speed of 10 rpm. The microarray slide was washed with gene expression wash buffers provided by Agilent Technologies, followed by an acetonitrile wash to avoid sediment and dry the slide. Agilent SureScan was used to scan the microarray slide.

### Microarray data analysis

Data were extracted using Agilent Feature Extraction software. The fluorescence intensity for each spot was corrected by subtracting the background using the standard Feature Extraction algorithm. Then, data was normalized using Lowess algorithm, which is applied for two-color data to compensate dye bias incorporation. The metrics parameters were considered adequate. GeneSpring GX software (Agilent Technologies, Santa Clara, CA, USA) was used for differential gene expression analysis. Significant changes were defined using t test against zero on the basis of fold change (≥ 2) and p value (≤ 0.05) cut offs. The log ratio (log two fold change) was taken. The functional significance of the genes with differential expression was determined in Ingenuity Pathway Analysis software (IPA; Qiagen, Valencia, CA, USA) by right-tailed Fisher’s exact test with p value of overlap ≤ 0.05. The z score (≥ 2 or ≤ − 2) was considered.

## Supplementary Information

Supplementary Information 1.
