# Supplementary material for: Stress-induced differential gene expression in cardiac tissue
Source: Sci Rep. 2021 Apr 28;11:9129. doi: 10.1038/s41598-021-88267-8 (PMC8080723; doi:10.1038/s41598-021-88267-8)
Supplement: Supplementary file 1 — Supplementary Information 1. [file 41598_2021_88267_MOESM1_ESM.pdf]

Stress-induced differential gene expression in cardiac tissue

Ana Elisa T.S. de Carvalho*, Marco A. Cordeiro, Luana S. Rodrigues, Daniela Ortolani, Regina C. Spadari*

Laboratory of Stress Biology, Department of Biosciences, Institute of Health and Society, Campus Baixada Santista, Federal University of São Paulo (UNIFESP), Santos, São Paulo, Brazil

Authors for correspondence: R.C. Spadari and A.E.T.S. de Carvalho
Laboratório de Biologia do Estresse
Departamento de Biociências
ISS - Campus Baixada Santista
Universidade Federal de São Paulo (UNIFESP)
Rua Silva Jardim, 136, sala 310
Santos, São Paulo
CEP 11020-015
Brazil
email: regina.spadari@unifesp.br
aetscarvalho@unifesp.br
Phone number: +55 (13) 32290194

Supplementary Table S1: The disregulated genes identified in the heart by the Ingenuity Pathway Analysis based on the comparison of stressed with non-stressed untreated rats (4 rats/group). The disregulated genes were ranked alphabetically and thresholds were based on extent of fold change (≥ 2) and p-value (≤ 0.05).

Symbol	Gene name	Expr p-value	Expr Log ratio
Abcg3	ATP binding cassette, subfamily G, member 3	2.65E-02	1.824
ACBD4	acyl-CoA binding domain containing 4	4.00E-02	1.319
ACOXL	acyl-CoA oxidase like	4.47E-03	1.136
ADAMTS1	ADAM metallopeptidase with thrombospondin type 1 motif, 1	4.22E-03	1.00
ADAMTSL2	ADAMTS like 2	3.62E-02	1.069
Ahsa2	AHA1, activator of heat shock protein	3.61E-02	1.243
ALAD	aminolevulinate	4.63E-02	-1.268
ANXA3	annexin A3	3.18E-02	1.688
APLN	apelin	1.50E-02	-1.119
APOL2	apolipoprotein L2	1.43E-02	1.34
ARL4A	ADP-ribosylation factor like GTPase 4A	1.29E-03	1.388
Arl5b	ADP ribosylation factor like GTPase 5B	3.58E-03	1.057
BFAR	bifunctional apoptosis regulator	6.89E-03	1.091
BTG2	BTG anti-proliferation factor 2	6.99E-03	1.335
BTG3	BTG anti-proliferation factor 3	6.81E-03	1.347
C1QTNF12	C1q and TNF related 12	2.84E-02	1.062
C2CD4A	C2 calcium dependent domain containing 4A	1.77E-02	-1.634
C2orf40	chromossome 2 open	1.57E-02	-1.035
C9orf85	chromossome 9 open	2.56E-02	-1.019
CBY1	chibby family member 1	4.32E-02	-1.203
CCL4	C-C motif chemokine ligand 4	1.62E-02	-1.11

CCNQ	cyclin Q	3.36E-02	-1.163
CDK1	cyclin dependent kinase 1	7.51E-03	-1.142
CDKN2C	cyclin dependent kinase inhibitor 2C	1.12E-03	-1.554
CEBPD	CCAAT enhancer binding protein delta	1.13E-02	1.079
CEL	carboxyl ester lipase	4.95E-02	1.242
CEP350	centrossomal protein 350	1.33E-02	-1.028
CFAP57	cilia and flagella associated protein 57	1.83E-02	-1.037
CHMP1B	charged multivesicular body protein 1B	1.06E-02	-1.014
CHRDL1	chordon like 1	3.18E-02	1.107
CIRBP	cold inducible RNA binding protein	3.92E-02	1.157
CLDN22	claudin 22	1.82E-02	-1.058
COL7A1	collagen type VII alpha 1	2.43E-02	1.07
COMMD5	COMM domain containing 5	1.25E-02	-1.12
CPEB2	cytoplasmic polyadenylation element binding protein 2	1.15E-03	1.003
CPOX	coproporphyrinogen oxidase	8.76E-03	-1.133
CREM	cAMP responsive element modulator	3.77E-03	1.933
CRISPLD1	cysteine-rich secretory protein LCCL domain containing 1	4.65E-02	1.351
CTNS	cystinosin, lysosomal cystine transporter	2.98E-02	1.177
DBI	diazepam binding inhibitor	1.93E-02	1.036
Ddx3	DEAD (Asp-Glu-Ala-Asp)	3.70E-03	1.087
DYRK3	dual specificity tyrosine phosphorylation regulated kinase 3	2.06E-02	1.745
EGR1	early growth response 1	2.27E-02	2.087
EIF2D	eukaryotic translation initiation factor 2D	3.50E-02	1.309
ELL2	elongation factor for RNA polymerase II 2	8.08E-03	1.716
ERRFI1	ERRBB receptor feedback inhibitor 1	6.64E-04	1.801

FAM167b	family with sequence similarity 167, member B	1.08E-02	-1.023
FOSL2	FOS like 2, AP-1	2.57E-02	1.73
FSIP1	fibrous sheath interacting protein 1	3.59E-02	1.311
GNAT1	G protein subunit alpha transducin 1	7.35E-03	1.705
GPATCH3	G-patch domain containing 3	1.15E-02	-1.068
GPR183	G protein coupled receptor 183	2.35E-02	-1.048
GPRC5D	G protein coupled receptor, class C, group 5, member D	2.68E-03	1.295
GRAMD1C	GRAM domain containing 1C	4.14E-02	1.326
GRHL1	grainyhead like transcription factor 1	4.66E-02	1.00
GSTA5	glutathione S-transferase alpha 5	1.38E-02	-1.534
GZMA	granzyme A	6.22E-04	-1.007
HAS1	hyaluronan synthase 1	1.93E-02	1.429
HDAC8	histone deacetylase 8	3.92E-02	1.321
HPS4	biogenesis of lysosomal organelles complex 3 subunit 2	2.60E-03	1.489
HSD17B12	hydroxysteroid 17-beta dehydrogenase 12	2.39E-02	-1.068
HSP90AA1	heat shock protein 90 alpha family class A member 1	3.37E-02	1.38
Humwe1	HECT, UBA and WWE	4.47E-02	1.179
IBA57	iron-sulfur cluster assembly factor IBA57	5.00E-02	1.352
IER2	immediate early response 2	5.28E-03	1.071
Ifi47	interferon gamma inducible protein 47	2.21E-02	-1.307
IFRD1	interferon related developmental regulator 1	6.21E-06	1.297
IL22RA2	interleukin 22 receptor subunit alpha 2	2.31E-02	1.386
IL2RA	interleukin 2 receptor subunit alpha	1.62E-02	1.247
IL6R	interleukin 6 receptor	1.68E-02	1.923

KLF6	Kruppel like factor 6	7.66E-03	1.366
LOC100909409* (includes others)	RGD1562660	2.01E-02	1.909
LOC1009102237*	uncharacterized	1.60E-02	-1.055
LOC100911553*	uncharacterized	3.50E-02	1.081
LOC102547300*	uncharacterized	3.14E-02	1.159
LOC102548125*	uncharacterized	3.64E-04	1.22
LOC102549311*	uncharacterized	2.72E-02	1.292
LOC102550392*	uncharacterized	5.32E-03	1.023
LOC102550730*	uncharacterized	3.19E-02	1.274
LOC102550980*	uncharacterized	3.29E-02	1.007
LOC102551486*	uncharacterized	2.40E-02	2.572
LOC102551514*	uncharacterized	2.26E-02	1.31
LOC102552452*	uncharacterized	2.66E-02	1.247
LOC102552654*	uncharacterized	4.11E-02	1.019
LOC102553678*	uncharacterized	4.31E-02	1.414
LOC102554725*	uncharacterized	3.79E-02	1.145
LOC102555894*	uncharacterized	6.84E-04	1.128
LOC102556325*	uncharacterized	4.74E-02	1.157
LOC102556393*	uncharacterized	2.21E-02	1.248
LOC102556673*	uncharacterized	9.28E-03	3.327
LOC103690351*	uncharacterized	3.22E-02	1.141
LOC103690394*	uncharacterized	4.72E-02	1.336
LOC103690486*	uncharacterized	4.84E-02	1.318
LOC103690934*	uncharacterized	2.67E-02	1.811
LOC103691866*	uncharacterized	3.52E-02	2.21
LOC103693175*	uncharacterized	2.56E-02	1.966
LOC103694115*	ankyrin repeat domain	2.95E-02	1.375

LOC103694161*	uncharacterized	4.64E-02	1.374
LOC103695196*	uncharacterized	4.68E-03	-1.074
LOC108348225*	feline leukemia virus	1.07E-02	1.045
LOC108348293*	uncharacterized	2.10E-02	1.784
LOC108350833*	uncharacterized	3.35E-02	1.039
LOC10099409* (includes others)	RGD1562660	2.01E-02	1.909
MAP3K6	mitogen-activated protein kinase kinase kinase 6	1.26E-02	1.622
MAT2A	methionine adenosyltransferase 2A	2.17E-02	1.506
MLF1	myeloid leukemia factor 1	1.74E-02	-1.15
MLXIPL	MLX interacting protein like	9.67E-03	1.003
MTHFR	methylenetetrahydrofolate reductase	9.22E-03	1.624
MYBPC2	myosin binding protein C2	3.52E-02	-1.399
MYBPH	myosin binding protein H	3.78E-02	1.083
MYCN	MYCN proto-oncogene	4.37E-03	-2.309
NOCT	nocturnin	8.93E-03	1.399
OLAH	oleoyl-ACP-hydrolase	2.28E-02	-1.073
P2RY1	purinergic receptor P2Y1	1.01E-02	-1.076
PARP12	poly(ADP-ribose) polymerase family, member 12	3.76E-02	1.002
PAX3	paired box 3	3.32E-02	1.284
PDLIM3	PDZ and LIM domain 3	3.28E-03	2.168
PFKB1	6-phospofructo-2-kinase	3.50E-02	1.59
PHF20L1	PHD finger protein 20 like 1	3.76E-02	1.093
PLA2G2F	phospholipase A2 group IIF	6.22E-03	1.521
PLAUR	plasminogen activator, urokinase receptor	2.46E-03	1.703
PLK3	polo like kinase 3	1.24E-02	1.629
PNMA3	PNMA family member 3	4.47E-02	1.255

POF1B	POF1B actin binding protein	3.84E-02	1.141
PPP1CB	protein phosphatase 1, catalytic subunit beta	1.94E-02	-1.114
Ppp1r15a	protein phosphatase 1 , regulatory subunit 15A	3.69E-03	1.128
PRDM1	PR/SET domain 1	4.31E-03	-1.069
PSPN	persephin	1.98E-02	1.064
PTGDS	prostaglandin D2 synthase	1.94E-02	-1.458
PTGDR	prostagladin F receptor	2.58E-02	1.025
RGD1306750*	LOC362451	9.66E-03	-1.117
RGD1560523	similar to S-adenosylmethionine synthetase gamma form	2.83E-02	1.506
RGS1	regulator of G protein signaling 1	9.66E-03	2.489
RGS16	regulator of G protein signaling 16	2.13E-04	1.196
RHOB	ras homolog family member B	3.46E-02	1.249
ROS1	ROS proto-oncogene 1	3.01E-02	1.037
Rpl22l1	ribosomal protein L22 like 1	4.39E-02	-1.116
RRAD	RRAD, Ras related glycolysis inhibitor and calcium channel regulator	4.57E-02	-1.41
SAT1	spermidine/spermine N1	1.91E-03	1.146
SIGLEC10	sialic acid binding Ig like lectin 10	2.90E-02	1.025
SIMC1	SUMO interacting motifs containing 1	2.58E-02	1.511
SLC1A1	solute carrier family 1	2.61E-03	1.145
SLC20A1	solute carrier family 20	2.98E-02	1.167
SLC25A25	solute carrier family 25	1.96E-03	1.459
SLC25A30	solute carrier family 25	1.86E-02	1.117
SLC7A6	solute carrier family 7	2.19E-02	1.125
SNED1	Sushi, nidogen and EGF-like domain 1	3.66E-02	1.084
SNRPD1	small nuclear ribonucleoprotein D1 polypeptide	7.76E-04	1.198

SOX18	SRY-box 18	1.20E-03	-1.241
STX1A	syntaxin 1A	3.28E-03	1.051
SUPT3H	SPT3 homolog, SAGA and STAGA complex component	4.25E-02	1.411
TAMM41	TAM41 mitochondrial translocator assembly and maintenance homolog	2.80E-02	-1.021
TARBP1	TAR (HIV-1) RNA binding protein 1	4.61E-02	1.201
TBC1D7	TBC1 domain family member 7	1.54E-02	-1.007
TLR3	toll like receptor 3	4.73E-02	-1.402
TMEM196	transmembrane protein 196	2.66E-02	-1.183
TMEM255A	transmembrane protein 255A	6.06E-03	-1.142
TP53RK	TP53 regulating kinase	5.65E-03	1.559
TRIL	TLR4 interactor with leucine -rich repeats	1.26E-02	-1.025
TRIM17	tripartite motif containing 17	2.05E-02	1.353
TRPT1	tRNA phosphotransferase 1	4.88E-02	1.252
TSTD2	thiosulfate sulfurtransferase like domain containing 2	2.23E-02	1.131
TTC30B	tetratrocopeptode repeat domain 30B	1.39E-02	-1.366
WIF1	WNT inhibitor factor 1	1.34E-02	-1.141
WT1	Wilms tumor 1	2.89E-02	1.277
ZC3H12A	zinc finger CCCH-type containing 12A	1.56E-02	1.00
ZNF763	zinc finger protein 763	3.37E-03	-1.153
ZNF627	zinc finger protein 627	3.64E-03	-1.061

*non-annotated genes

Supplementary Table S2: The disregulated genes identified in the heart by the Ingenuity Pathway Analysis based on the comparison of those stressed and unstressed rats treated with ICI118,551 (4 rats/group). The disregulated genes were ranked alphabetically and thresholds were based on extent of fold change (≥ 2) and p-value (≤ 0.05).

Symbol	Gene name	Expr p-value	Expr Log Ratio
Aph1c	aph 1 homolog C, gamma secretase subunit	3.14E-02	1.147
APLNR	apelin receptor	9.40E-03	-1.31
APOLD1	apolipoprotein L domain 1	1.88E-03	2.195
ARC	activity regulated cytoskeleton-associated protein	2.35E-02	1.518
ARNTL	aryl hydrocarbon receptor nuclear translocator-like	4.07E-02	-1.238
ART5	ADP-ribosyltransferase 5	3.91E-02	-1.146
ATF3	activating transcription factor 3	3.52E-02	1.814
Bex1/LOC100912195	Brain´s expressed, X-linked	1.88E-02	1.014
BUB1	BUB1 mitotic checkpoint serine/threonine kinase	2.30E-02	-1.038
CCL2	C-C motif chemokine ligand 2	7.14E-03	-2.171
CEBPB	CCAAT enhancer binding protein beta	2.00E-03	1.407
CEP295	centrosomal protein 295	3.32E-02	-1.141
CLDN19	claudin 19	4.32E-02	1.164
COQ10B	coenzyme Q10B	3.88E-03	1.512
Cpa6	carboxypeptidase A6	1.63E-02	1.611
CREM	cAMP responsive element modulator	1.92E-03	1.005
CRYL1	crystallin lambda 1	3.25E-02	1.3

CSGALNACT1	chondroitin sulfate N-acetylgalactosaminyltransferase 1	4.18E-02	1.0977
CSRNP1	cysteine and serine rich nuclear protein 1	2.03E-03	1.00
DDIT4	DNA damage inducible transcript 4	1.97E-02	1.067
DNAJA1	DnaJ heat shock protein family (Hsp40) member A1	3.70E-02	1.543
DNAJB1	DnaJ heat shock protein family (Hsp40) member B1	3.63E-02	1.605
DNAJB4	DnaJ heat shock protein family (Hsp40) member B4	3.40E-02	1.219
DUSP1	dual specificity phosphatase 1	4.37E-02	1.786
ENC1	ectodermal-neural cortex 1	8.95E-03	1.51
ERRFI1	ERBB receptor feedback inhibitor 1	1.50E-02	1.06
Gstt3	glutathione S-transferase, theta 3	7.14E-03	-1.01
Hist1h1b	histone cluster 1 H1b	4.21E-03	-1.684
HJURP	Holliday junction recognition protein	3.20E-02	-1.016
HSP90AA1	heat shock protein 90 alpha family class A member 1	4.37E-02	1.836
HSPA1A/HSPA1B	heat shock protein family A (Hsp70) member 1A/1B	2.79E-02	3.867
IRS2	insulin receptor substrate 2	1.47E-02	2.132
ITGB3BP	integrin subunit beta 3 binding protein	4.66E-02	1.59
IVNS1ABP	influenza virus NS1A binding protein	3.86E-02	1.362
JUN	Jun proto-oncogene	1.52E-02	1.478
KIF22	kinesin family member 22	4.58E-02	-1.319
KLF2	Kruppel like factor 2	1.42E-03	1.577
KLF4	Kruppel like factor 4	6.87E-03	1.108

LOC100909782*	uncharacterized	1.93E-02	1.313
LOC100910949*	uncharacterized	2.64E-02	1.528
LOC100911299*	uncharacterized	2.08E-02	-1.107
LOC102546354/Zfp14	ZFP14 zinc finger protein	4.33E-02	1.091
LOC102548611*	uncharacterized	1.05E-02	-1.277
LOC102549227*	uncharacterized	3.15E-02	1.379
LOC102553140*	uncharacterized	1.58E-02	-1.001
LOC102555914*	tRNA-splicing	7.76E-03	1.33
LOC103690476*	uncharacterized	3.85E-02	-1.129
LTC4S	leukotriene C4 synthase	3.79E-03	1.237
LYVE1	lymphatic vessel endothelial hyaluronan receptor 1	1.14E-02	1.04.
MAP2K3	mitogen activated protein kinase kinase 3	4.87E-02	1.072
MCA4R	melanocortin 4 receptor	3.18E-02	1.523
Ms4a6b	membrane-spanning 4-domains, subfamily A, member 6B	4.76E-02	1.027
MYL1	myosin, light chain 1	1.42E-02	1.245
Nat8f3 (include others)	N-acetyltransferase 8 (GCN5-related) family member 3	9.61E-04	1.197
NFKBIA	NFKB inhibitor alpha	6.83E-04	1.252
NPAS2	neuronal PAS domain protein 2	2.10E-02	-1.037
NR4A1	nuclear receptor subfamily 4, group A, member 1	4.16E-03	1.772
NR4A2	nuclear receptor subfamily 4, group A, member 2	1.33E-02	1.22
OTUD1	OTU deubquitinase 1	3.57E-02	1.106
PDK4	pyruvate dehydrogenase kinase 4	3.12E-02	1.335

PER1	period circadian regulator 1	7.78E-03	2.036
PER2	period circadian regulator 2	3.65E-02	1.409
PM20D2	peptidase M20 domain 2	4.14E-02	-1.341
PPM1K	protein phosphatase, Mg2+/Mn2+ dependent, 1K	1.35E-02	1.087
RASSF1	Ras association domain family member 1	1.21E-03	1.109
S100A8	S100 calcium binding protein A8	3.20E-02	-1.005
S100A9	S100 calcium binding protein A9	3.43E-02	-1.13
SERPINE1	serpin family E member 1	2.96E-02	1.531
SEZ6L	seizure related 6 homolog like	1.11E-03	-2.84
SGCB	sarcoglycan beta	4.27E-02	-1.904
SGK1	serum/glucocorticoid regulated kinase 1	2.86E-02	1.055
Sik1	salt inducible kinase 1	8.91E-04	2.041
SPAG5	sperm associated antigen 5	4.82E+02	-1.052
STC1	stanniocalcin 1	1.26E-02	1.828
Tcap	titin-cap	1.40E-02	1.013
TRIM21	tripartite motif -containing 21	2.99E-02	2.029
Tsc22d3	TSC22 domain family member 3	1.30E-03	1.058
USP2	ubiquitin specific peptidase 2	4.97E-02	1.907
ZBTB16	zinc finger and BTB domain containing 16	7.20E-03	1.102

*non-annotated genes

Supplementary Table S3: Diseases and functions identified in the heart by the Ingenuity Pathway Analysis based on the comparison of stressed with non-stressed untreated rats (4 rats/group), and those stressed and unstressed treated with ICI118,551 (4 rats/group). The thresholds were z-score (≥ 2 or ≤ -2) and p-value (≤ 0.05).

		ST			
Categories	Diseases or Functions Annotation	p-value	Predicted Activation State	z-score	Molecules
Cell Death and Survival, Hair and Skin Development and Function	Cell viability of epithelial cell lines	7.38E-03	Increased	2.000	BTG2, CEBPD, DYRK3, WT1
Cardiovascular System Development and Function	Development of vasculature	1.10E-02	Increased	2.041	ADAMTS1, ANXA3, APLN, EGR1, ERRFI1, HAS1, HSP90AA1, IL6R, KLF6, MTHFR, MYCN, PLAUR, Ppp1r15a, PRDM1, RHOB, SOX18, TLR3, WT1, ZC3H12A
Cardiovascular System Development and Function, Organismal Development	Angiogenesis	1.25E-02	Increased	2.041	ADAMTS1, ANXA3, APLN, EGR1, HAS1, HSP90AA1, IL6R, KLF6, MTHFR, PLAUR, Ppp1r15a, PRDM1, RHOB, SOX18, TLR3, WT1, ZC3H12A
Cellular Development, Cellular Growth and Proliferation	Cell proliferation of lymphoma cell lines	1.31E-02	Increased	2.162	EGR1, HDAC8, IL6R, PRDM1, WT1
DNA Replication, Recombination, and Repair, Nucleic Acid Metabolism, Small Molecule Biochemistry	Incorporation of thymidine	5.39E-04	Decreased	-2.000	APLN, CREM, PAX3, SAT1, WT1
Organismal Injury and Abnormalities	Interstitial fibrosis	1.13E-03	Decreased	-2.000	EGR1, IL2RA, P2RY1, PPP1CB, WT1

Cell Death and Survival	Apoptosis of lymphoid organ	1.03E-02	Decreased	-2.183	EGR1, GZMA, IL6R, MLF1, WT1

ICIST

Categories	Diseases or Functions Annotation	p-value	Predicted Activation State	z-score	Molecules
Cellular Function and Maintenance	Cellular homeostasis	8.89E-06	Increased	2.742	ARNTL, CCL2, CEBPB, DDIT4, DNAJB1, HSP90AA1, IRS2, JUN, KLF2, KLF4, MC4R, NFKBIA, NR4A1, NR4A2, PDK4, S100A8, S100A9, SERPINE1, SGK1, SIK1, STC1, TRIM21, ZBTB16
Organismal Survival	Survival of organism	1.92E-03	Increased	2.345	CCL2, CEBPB, DUSP1, HSPA1A/HSPA1B, MYL1, NFKBIA, PER2, RASSF1, S100A9, SERPINE1, USP2
Reproductive System Development	Fertility	1.31E-05	Increased	2.343	CEBP, CREM, DNAJA1, HSP90AA1, IRS2, MC4R, PER1, PER2, STC1, USP2
Energy Production, Lipid Metabolic	Oxidation of fatty acid	1.45E-03	Increased	2.2	IRS2, MC4R, NR4A1, NR4A2, PDK4
Lipid Metabolism, Molecular Transport, Small Molecule Biochemistry	Efflux of cholesterol	9.56E-05	Increased	2.194	CCL2, MAP2K3, NFKBIA, S100A8, S100A9
Embryonic Development, Organ Development, Organismal Development	Development of body trunk	6.66E-04	Increased	2.144	Aph1c, APLNR, ARNTL, ATF3, CCL2, CEBPB, DUSP1, ERRFI1, IRS2, JUN, KLF2, KLF4, MC4R, NR4A1, RASSF1, SERPINE
Cell Death and Survival	Cell viability	4.52E-07	Increased	2.052	ATF3, GCCL2, CEBPB, DUSP1, HSPA1A/HSPA1B, IRS2, JUN, KLF2, KLF4, MAP2K3, NFKBIA, NR4A1, NR4A2, PDK4, RASSF1, S100A8, S100A9, SERPINE1, SGK1, STC1, TRIM21, USP2, ZBTB16

Organismal Survival	Organismal death	1.54E-06	Decreased	-2.372	APLNR, ARC, ARNTL, ATF3, BUB1, CCL2, CEBPB, CREM, CSGALNACT1, CSRNP1, DNAJB1, DNAJB4, DUSP1, ERRFI1, HSP90AA1, IRS2, IVNS1ABP, JUN, KIF22, KLF2, KLF4, MAP2K3, NFKBIA, NR4A2, PER2, RASSF1, S100A8, S100A9, SERPINE1, SIL1, TRIM21
Organismal Injury and Abnormalities	Urination disorder	1.61E-04	Decreased	-2.0	APLNR, CCL2, IRS2, PPM1K, SERPINE1, SGK1, STC1, TRIM21

Supplementary Table S4: Canonical pathways analysis identified in the heart by the Ingenuity Pathway Analysis based on the comparison of stressed with non-stressed untreated rats (4 rats/group), and those stressed and unstressed treated with ICI118,551 (4 rats/group). The thresholds were z-score (≥ 2 or ≤ -2) and p-value (≤ 0.05).

	ST		
Canonical pathway	p-value	Predicted Activation State by z-score	Molecules
Heme biosynthesis II	1.88E-03	Zero	ALAD, CPOX
Eicosanoid signaling	9.45E-03	Zero	PLA2G2F, PTGDS, PTGFR
Mitotic roles of polo-like kinase	9.90E-03	Zero	CDK1, HSP90AA1, PLK3
Cyclins and cell cycle regulation	1.74E-02	Zero	CDK1, CDKN2C, HDAC8
S-adenosyl-L-methionine biosynthesis	2.20E-02	Zero	MAT2A
Cell cycle regulation by BTG family proteins	2.46E-02	Zero	BTG2, NOCT
Heme biosynthesis from uroporphyrinogen III I	2.92E-02	Zero	CPOX
Spermine and Spermidine degradation I	2.92E-02	Zero	SAT1

Tetrapyrrole biosynthesis II	3.64E-02	Zero	ALAD
Telomerase signaling	3.69E-02	Zero	HDAC8, HSP90AA1, IL2RA

ICIST

Categories	p-value	Predicted Activation State by z-score	Molecules
Aldosterone signaling in epithelial cells	1.99E-06	Zero	DNAJA1, DNAJB1, DNAJB4, DUSP1, HSP90AA1, HSPA1A/HSPA1B, SGK1
Glucocorticoid receptor signaling	2.32E-06	No activation	CCL2, CEBPB, DUSP1, HSP90AA1, HSPA1A/HSPA1B, JUN, NFKBIA, SERPINE1, SGK1
IL17A signaling in fibroblasts	7.43E-06	No activation	CCL2, CEBPB, JUN, NFKBIA
IL17 signaling	2.10E-04	No activation	CCL2, CEBPB, JUN, MAP2K3
Circadian rhythm signaling	2.71E-04	No activation	ARNTL, PER1, PER2
Role of IL17A in psoriasis	3.21E-04	No activation	S100A8, S100A9
Protein ubiquitination pathway	4.05E-04	No activation	DNAJA1, DNAJB1, DNAJB4, HSP90AA1, HSPA1A/HSPA1B, USP2
Acute phase response signaling	5.06E-04	Positive	CEBPB, JUN, MAP2K3, NFKBIA, SERPINE1
Role of IL17A in arthritis	9.61E-04	No activation	CCL2, MAP2K3, NFKBIA

Apelin endothelial signaling pathway	1.01E-03	Zero	APLNR, CCL2, JUN, KLF2
IL6 signaling	1.39E-03	Positive	CEBPB, JUN, MAP2K3, NFKBIA
PI3K signaling in B lymphocytes	1.91E-03	Zero	ATF3, RS2, JUN, NFKBIA
CD27 signaling in lymphocytes	1.08E-03	Zero	JUN MAP2K3, NFKBIA
CD40 signaling	1.99E-03	Zero	JUN MAP2K3, NFKBIA
IL10 signaling	2.27E-03	No activation	JUN MAP2K3, NFKBIA
cAMP mediated signaling	1.06E-02	Negative	APLNR, CREM, DUSP1, MC4R

Supplementary Table S5: The upstream regulators identified in the heart by the Ingenuity Pathway Analysis based on the comparison of stressed with non-stressed untreated rats (4 rats/group), and those stressed and unstressed treated with ICI118,551 (4 rats/group). The thresholds were z-score (≥ 2 or ≤ -2) and p-value (≤ 0.05).

		ST		
Upstream regulator	name	z-score	Predicted Activation State	p-value
Forskolin	forskolin	3.115	Activated	5.72E-06
TNF	tumor necrosis factor	3.088	Activated	7.32E-07
IL1B	interleukin 1 beta	2.967	Activated	8.07E-10
Doxorubicin	doxorubicin	2.761	Activated	7.91E-03
F2	coagulation factor II, thrombin	2.757	Activated	4.20E-04
CG	complex	2.701	Activated	4.0E-05
Ca+2	calcium	2.586	Activated	1.80E-03
4-hydroxyltamoxifen	4-hydroxyltamoxifen	2.433	Activated	3.16E-05

GPER1	G protein-coupled estrogen receptor 1	2.425	Activated	8.28E-07
Cisplatin	cisplatin	2.424	Activated	2.15E-02
MAPK1	mitogen-activated protein kinase 1	2.414	Activated	2.60E-03
MAPK8	mitogen-activated protein kinase 8	2.391	Activated	9.69E-04
Gentamicin	gentamicin	2.333	Activated	9.39E-05
SMARCA4	SWI/SNF related, matrix associated, actin dependent regulator of chromatin, subfamily a, member 4	2.213	Activated	4.45E-02
TGFB1	transforming growth factor beta 1	2.206	Activated	3.51E-05
Isobutylmethylxanthine	isobutylmethylxanthine	2.190	Activated	1.50E-02
Insulin	insulin	2.184	Activated	1.14E-02
PGR	progesterone receptor	2.177	Activated	1.38E-02
Kainic acid	kainic acid	2.148	Activated	4.86E-03
CREB1	cAMP responsive element binding protein 1	2.140	Activated	5.93E-03
Tetradecanoylphorbol acetate	tetradecanoylphorbol acetate	2.126	Activated	2.76E-07

EGF	epidermal growth factor	2.078	Activated	5.53E-04
bucladesine	bucladesine	2.013	Activated	9.83E-04
NR3C2	nuclear receptor subfamily 3 group C member 2	2.000	Activated	1.33E-02
Camptothecin	camptothecin	2.000	Activated	1.96E-02
PD98059	MAP kinase inhibitor	-2.427	Inhibited	1.11E-04
ACOX1	acyl-CoA oxidase 1	-2.000	Inhibited	1.87E-02

ICIST

Upstream regulator	name	z-score	Predicted Activation State	p-value of overlap
CREB1	cAMP responsive element binding protein 1	3.634	Activated	1.40E-12
Kainic acid	kainic acid	3.23	Activated	5.08E-13
Dexamethasone	dexamethasone	3.155	Activated	6.15E-08
forskolin	forskolin	3.077	Activated	1.92E-16
Insulin	insulin	3.035	Activated	1.15E-15
NUPR1	nuclear protein 1, transcriptional regulator	3.0	Activated	6.56E-05

PGR	progesterone receptor	2.96	Activated	6.53E-09
methylprednisolone	methylprednisolone	2.817	Activated	1.36E-07
bucladesine	bucladesine	2.795	Activated	8.36E-09
GPER1	G protein-coupled estrogen receptor 1	2.646	Activated	3.89E-10
dalfampridine	dalfampridine	2.646	Activated	1.27E-10
Dexamethasone phosphate	dexamethasone phosphate	2.63	Activated	4.45E-12
ionomycin	ionomycin	2.607	Activated	4.21E-06
GnRH-A	gonadotropin-releasing hormone analog	2.607	Activated	8.75E-09
HIF1A	hypoxia inducible factor 1 subunit alpha	2.574	Activated	7.44E-04
Bicuculline	bicuculline	2.56	Activated	5.45E-10
Norepinephrine	norepinephrine	2.553	Activated	1.43E-12
Hydrogen peroxide	hydrogen peroxide	2.528	Activated	8.19E-10

EGF	epidermal growth factor	2.459	Activated	2.17E-08
Decitabine	decitabine	2.425	Activated	1.70E-03
2-amino-5-phosphonoval	2-amino-5-phosphonoval	-2.425	Inhibited	1.29E-05
PD98059	MAP kinase inhibitor	-4.42	Inhibited	9.21E08
U0126	MAP kinase inhibitor	-2.299	Inhibited	1.99E-11
ESR1	estrogen receptor alpha	-2.043	Inhibited	2.09E-04
NKX2-3	NK2 Homeobox 3	-2.000	Inhibited	6.88E-03
